# Glycosylation Variants of a β-Glucosidase Secreted by a Taiwanese Fungus, *Chaetomella raphigera*, Exhibit Variant-Specific Catalytic and Biochemical Properties

**DOI:** 10.1371/journal.pone.0106306

**Published:** 2014-09-02

**Authors:** Aki Yoneda, Hsion-Wen David Kuo, Mayumi Ishihara, Parastoo Azadi, Su-May Yu, Tuan-hua David Ho

**Affiliations:** 1 Department of Biology, Washington University in St. Louis, St. Louis, Missouri, United States of America; 2 Department of Pathology and Immunology, School of Medicine, Washington University in St. Louis, St. Louis, Missouri, United States of America; 3 Department of Environmental Science and Engineering, Tunghai University, Taichung, Taiwan, Republic of China; 4 Complex Carbohydrate Research Center, University of Georgia, Athens, Georgia, United States of America; 5 Institute of Molecular Biology, Academia Sinica, Nankang, Taipei, Taiwan, Republic of China; 6 Agricultural Biotechnology Center, National Chung-Hsing University, Taichung, Taiwan, Republic of China; 7 Department of Life Sciences, National Chung-Hsing University, Taichung, Taiwan, Republic of China; 8 Institute of Plant and Microbial Biology, Academia Sinica, Nankang, Taipei, Taiwan, Republic of China; Russian Academy of Sciences, Institute for Biological Instrumentation, Russian Federation

## Abstract

Cellulosic biomass is an abundant and promising energy source. To make cellulosic biofuels competitive against conventional fuels, conversion of rigid plant materials into sugars must become efficient and cost-effective. During cellulose degradation, cellulolytic enzymes generate cellobiose (β-(1→4)-glucose dimer) molecules, which in turn inhibit such enzymes by negative feedback. β-Glucosidases (BGLs) cleave cellobiose into glucose monomers, assisting overall cellulolytic activities. Therefore, BGLs are essential for efficient conversion of cellulosic biomass into biofuels, and it is important to characterize newly isolated BGLs for useful traits. Here, we report our discovery that the indigenous Taiwanese fungus *Chaetomella raphigera* strain *D2* produces two molecular weight variants of a single BGL, D2-BGL (shortened to “D2”), which differ in *O*-glycosylation. The more extensively *O*-glycosylated form of native D2 (nD2L) has increased activity toward the natural substrate, cellobiose, compared to the less *O*-glycosylated form (nD2S). nD2L is more stable at 60°C, in acidic pH, and in the presence of the ionic detergent sodium dodecyl sulfate than nD2S. Furthermore, unlike nD2S, nD2L does not display substrate inhibition by an artificial substrate *p*-nitrophenyl glucopyranoside (pNPG), and the glucose feedback inhibition kinetics of nD2L is competitive (while it is non-competitive for nD2S), suggesting that these two glycovariants of D2 bind substrates differently. Interestingly, D2 produced in a heterologous system, *Pichia pastoris*, closely mimics properties of nD2S. Our studies suggest that *O*-glycosylation of D2 is important in determining its catalytic and biochemical properties.

## Introduction

Cellulosic biomass is an abundant and promising feedstock for next-generation biofuels in many countries [1]–[3]. Conventional biomass such as starch can be readily degraded to sugar monomers in an industrial scale. However, utilizing cellulosic biomass poses a major challenge: converting rigid and recalcitrant plant materials into simple fermentable sugars is not as easily nor economically achieved [4]. To make cellulosic biofuels, cellulosic biomass must be pretreated and efficiently converted to sugars [1]. Cellulose, the major component of plant biomass, is a rigid polymer of β-(1→4) linked (D)-glucose, which is further cross-linked by other polysaccharides and lignin. Pretreatment is usually required to make polysaccharide chains more accessible for enzymatic digestion [5]. To efficiently digest cellulose, three groups of cellulolytic enzymes must act together. Endo-β-(1→4)-glucanases digest cellulose from inner regions of cellulose chains, and exo-β-(1→4)-glucanases digest cellulose chains from the reducing or non-reducing ends [6]. These processes release oligosaccharides and cellobiose, a β-(1→4)-glucose dimer, which strongly inhibits most glucanase activities, but may be digested to glucose monomers by β-glucosidases (BGLs) [6]. BGLs are ubiquitous in nature, and are classified into glycosylhydrolase (GH) families 1 and 3 by the Carbohydrate-active enzymes database (CAZy, www.cazy.org). Most bacterial and fungal BGLs belong to the GH3 family, with specificities to myriad substrates [7].

Glycosylation is one of the most common post-translational modifications for eukaryotic proteins, influencing a number of protein properties and functions including folding, solubility, thermostability, and proteolytic resistance [8]–[11]. The two major types of protein glycosylation are *N*- and *O*-linked glycosylation. *N*-glycosylation begins in the ER, where an oligosaccharide attached to dolichol is flipped into the lumen of the ER, processed, transferred to a target arginine residue within a nascent polypeptide, and then further modified throughout the secretory pathway [12], [13]. *N*-glycan structures vary greatly among species, and some fungal species synthesize extremely large *N*-glycan moieties (∼200 mannose residues to two *N*-acetylglucosamine (GlcNAc) residues) [14]. The *O*-glycans, on the other hand, tend to be simpler. *O*-glycosylation is initiated by a transfer of a monosaccharide moiety from a UDP-sugar donor to a serine or threonine residue via hydroxyl linkage. In fungi, the first moiety to be transferred is generally mannose, and the modification is therefore termed *O*-mannosylation [15], [16]. The *N*-glycosylation is often required for catalytic activities of enzymes [17]–[19], and in some cases specific *N*-glycosylation sites involved in catalytic activities have been identified [20]–[22]. The effects of *O*-glycosylation on enzymatic properties are less understood, in part because investigating *O*-glycosylation of an active enzyme poses more challenges: a) There is a consensus site for *N*-glycosylation (asparagine-X-serine or threonine, where X is not a proline) [12], while there is less sequence consensus for *O*-glycosylation sites, although a number of prediction programs exist [23], b) chemical *O*-glycan removal results in protein degradation, and c) a lack of *O*-glycosylation probably affects *N*-glycosylation [24]. Nonetheless, there are some examples of *O*-glycosylation affecting protein functions. Naim and Lentz demonstrated that two forms of lactase-phlorizin hydrolase, one *N*-glycosylated and the other *N*- and *O*-glycosylated, hydrolyzed lactose with different reaction kinetics [25]. *O*-glycosylation may also be involved in structural changes [26], [27]. Recently, synthetic protein *O*-mannosylation technique resulting in homogeneously *O*-glycosylated proteins became available [28]. Chen *et al.* systematically showed that *O*-glycosylation enhances stability and substrate binding of model Family 1 carbohydrate-binding modules [29], suggesting that such modification would be advantageous for naturally produced enzymes.

In previous studies, Ng *et al.* isolated a native fungus, *Chaetomella raphigera* strain *D2*, from screening several dozens of wood-rotting fungi for BGL activities [30]. This fungus was identified by its ability to produce D2, a highly active BGL. The cDNA sequence encoding D2 was cloned into *Pichia pastoris* for a large-scale production. We noticed that *P. pastoris*-produced recombinant D2 (rD2) and native D2 isolated from *C. raphigera* have distinct catalytic and biochemical properties, and found that *C. raphigera* produces two forms of D2: a large, heavily glycosylated form (nD2L), and a small, *N*-glycosylated form (nD2S). In this study, we demonstrate similarities in properties of rD2 and nD2S, and how they differ from that of nD2L, suggesting that *O*-glycosylation can alter catalytic properties of *C. raphigera* BGL.

## Materials and Methods

### Cloning and sequencing of D2-BGL cDNA and production of recombinant D2-BGL

Since D2-BGL (shortened to “D2” hereafter) was the most abundant secretory protein from the fungus *C. raphigera* strain *D2*, it was easily purified by polyacrylamide gel electrophoresis (PAGE) followed by zymography (see Enzyme assays, (c) MUG in gel assay in Materials and Method). The N-terminal amino acid sequence of this protein was determined by Edman degradation to be N-PGDGDWAAA (carried out by Academia Sinica Protein Core Facility, Taipei). For D2 cDNA synthesis, total RNA isolated from actively growing *C. raphigera* strain *D2* was first reverse transcribed into single-stranded cDNA using a poly-T primer containing an anchor sequence (GGT TCT TGC CAC AGT CAC GAC TTT TTT TTT TTT TTT TTT), followed by PCR amplification of the cDNA copies using a forward primer (CCN GGN GAY GGN GAY TGG GC, designed based on N-terminal amino acid sequencing results) and an anchor as reverse primer (GGT TCT TGC CAC AGT CAC GAC). Invitrogen SuperScript III Reverse Transcriptase (Invitrogen, USA) was used in the reverse transcription and TaKaRa Ex Taq DNA Polymerase (TaKaRa Bio Inc, Japan) was used for PCR. The thermal cycling conditions were 94°C for 4 min, then 30 cycles of 94°C for 1 min, 58°C for 30 sec, and 72°C for 3 min, and a final extension at 72°C for 5 min.

The signal peptide sequence was cloned using inverse PCR. Briefly, genomic DNA (see genomic DNA isolation below) was digested with *Sac*I (New England Biolabs Inc., USA), self-ligated using T4 DNA ligase (Promega, USA), and then amplified by PCR using the primer pairs (233f: CGT TTC GTC CAA AAT GTA ACA GCA T and 232r: GAT GCT TTC ACC GTC AGT TCT GA). The thermal cycling conditions were 95°C for 5 min, then 25 cycles of 95°C for 1 min, 55°C for 1 min, and 72°C for 6 min, and a final extension at 72°C for 5 min. The inverse PCR product was cloned into the pGEM®-T Easy cloning vector and transformed into *Escherichia coli* DH5α.

Production of rD2 was accomplished by subcloning the D2-BGL cDNA into the vector pGAPZαC with the GAP promoter for constitutive expression in *P. pastoris*. A wild type strain SMD1168 was electroporated with the plasmid to allow production of rD2 with a C-terminal heptahistidine tag.

### Strains and fungal cultures

rD2-expressing *P. pastoris* strain was streaked on YPD (1% yeast extract, 2% peptone, 2% glucose) agar plates and routinely grown in YPD broth. *C. raphigera* was maintained on PDA (potato dextrose agar; Sigma #70139) plates. Spores were used to inoculate the center of plates, and allowed to grow at 30°C for 14 days. After 14 days, fungal biomass with agar from the plates were transferred to a flask containing 200-ml liquid medium (YPD or YP (1% yeast extract and 2% peptone) with or without the addition of 10 mM cellobiose or 20 mM glucose, depending on the experiment) for three days.

### Genomic DNA isolation of *C. raphigera* strain *D2*

Spores were used to inoculate 3-ml of YPD liquid medium in a test tube, and the culture was shaken at room temperature for three days. Then the fungal cells and hyphae were harvested by centrifugation in a 1.6-ml microtube, and 500 µl of lysis buffer (50 mM Tris-HCl pH 8, 170 mM EDTA, 1% SDS) and approximately 250 µl of 0.5 mm-glass beads (Biospec) were added to the tube. The tube was then agitated by vortex mixer for 2 min, cooled on ice for 1 min, and this step was repeated until cells appeared to have broken under a phase microscope. The tube was then heated at 65°C for 10 minutes. 300 µl of 7.5 M sodium acetate was added to the tube, and placed on ice for 30 min after thoroughly mixed by inverting the tube. The tube was then centrifuged at 17,000 × g for 10 minutes. To 700 µl of the supernatant transferred to a new microtube, 500 µl of isopropanol was added, and the mixture was placed on ice for 30 minutes. It was then centrifuged at 17,000 × g again to pellet DNA. The supernatant was discarded by aspiration, and 250 µl of TE was added to the resulting pellet for a 10-min incubation at 50°C. The pellet was dissolved by occasionally flicking the tube during the incubation. To the DNA solution, 200 µl of 24∶1 mixture of chloroform: isoamyl alcohol was added, mixed by inverting the tube, and centrifuged for 10 min. The top layer was transferred to a new microtube, and the genomic DNA was concentrated by ethanol precipitation. Concentration of DNA was determined using NanoDrop (Thermo Scientific).

### Southern blotting

DNA probe was generated by PCR using AYP40 (5′-TCC AAC ATC GAT GAT CGG) and AYP42 (5′-GGT CGT CGA CAA TAC AAG C) and Biotin Decalabel Kit (Fermentas), using both cDNA and genomic DNA as templates. *C. raphigera* genomic DNA was digested with restriction enzymes overnight, and was separated by 0.8% agarose gel electrophoresis. The gel was treated, transferred, cross-linked with UV light, pre-hybridized using standard Southern blotting methods. The pre-hybridized membranes were hybridized with the genomic or cDNA probes overnight in separate glass bottles, and the signals were detected using chemiluminescence reagents [31].

### Sample preparation and chromatography

*C. raphigera* spores were used to inoculate the center of potato dextrose agar plates. The fungus on plates was allowed to grow at 30°C for 14 days. After 14 days, agar (with the entire fungus) from the plates was transferred to a flask containing 200-ml liquid medium (1% yeast extract, 2% peptone, with or without the addition of 10 mM cellobiose or 20 mM glucose, depending on the experiment). The liquid culture was then shaken at room temperature for specified lengths of time, and the debris of agar was removed with a strainer and then centrifuged to pellet fungal cells and smaller debris. Supernatant was then filtered through a 0.45-μm filter and concentrated approximately 20-fold by centrifugation at 4,000 xg for 30 min or until desired volume was achieved, in Amicon Ultra centrifugal filter tubes (Millipore UFC903008, molecular weight cut off 30 kDa). The filtered, concentrated supernatant was then dialyzed against 10 mM Bis-Tris buffer pH 6.5 at 4°C for 3 days with daily buffer changes.

The dialyzed supernatant was then concentrated as described above to <10 ml, and was loaded to a manually-packed DEAE Sepharose Fast Flow (GE Healthcare) column, equilibrated with 10 mM Bis-Tris buffer pH 6.5. Approximately 3-ml (150 drops per tube) fractions were collected over 0-1 M NaCl continuous gradient at 0.5 ml/min with a peristaltic pump.

BGL activity was detected by pNPG and cellobiose assays. The activity peak was pooled and concentrated down to <250 µl, and loaded onto a manually poured Sephadex G 200 column, and the sample was run by gravity flow. 350 drops per tube (approximately 8 ml) were collected, and the activity was detected by pNPG and cellobiose assays. *P. pastoris*-produced D2 was purified similarly, since the recombinant protein did not bind to nickel columns despite possessing a heptahistidine tag.

### Enzyme assays

#### a) pNPG assay

Partially purified enzymes (10 µl, 10–100 µg/ml) in 10 mM Bis-Tris pH 6.5 was dispensed onto wells made with Parafilm and a PCR tube rack. 90 µl of 5 mM pNPG (*p*-nitrophenyl β-D-glucopyranoside, Sigma N7006) in 50 mM acetate buffer pH 5.0 (unless specified) was serially diluted two-fold in 90 µl 50 mM acetate buffer pH 5.0 in an 8-well PCR strip tubes per enzyme. Using a multichannel pipette, 10 µl of the prepared enzymes were transferred to pre-warmed serial dilutions of substrate, and incubated at 50°C for exactly 1 minute until the reaction was stopped by simultaneous additions of 100 µl 2% Na_2_CO_3_ in each well, and the 200 µl of resulting reaction solutions were transferred to a column of a flat-bottom 96-well plate. Once all reactions were stopped, the plate was read at 405 nm along with *p*-nitrophenol (pNP) standards using VersaMax plate reader (Molecular Devices). Assays were repeated at least five times in the same condition.

pNP stock solution was made by dissolving pNP (Fluka 1048) in water at 5 mM. Serially diluted pNP starting at 250 µM was used as standards. Glucose inhibition assays were performed using the same condition except for the addition of 0, 3, 6, 9 mM glucose in substrate buffers. To determine pH optima, the assays were performed in 10 mM HCl (pH 2.0), 50 mM acetate buffer (pH 2.6–6.0) or 50 mM phosphate buffer (pH 6.0–7.0). Since the reaction time was 1 min, pH would have minimal effect on protein stability.

Km was calculated by plotting substrate concentrations (mM pNPG) and initial velocity (mM pNP/sec/mg protein) and nonlinear least-squares fitted with Solver add-in on Microsoft Excel [32]. For substrate inhibition kinetics, we used a simple equation shown in File S1. Glucose Ki values on pNPG assays were determined by plotting substrate concentrations and initial velocity in double-reciprocal (Lineweaver-Burk) plot using Km and formula shown in File S1.

#### b) Cellobiose assay

Partially purified enzymes (3 µl, 10–100 µg/ml) in 10 mM Bis-Tris pH 6.5 was pipetted onto wells made with Parafilm and a PCR tube rack. 27 µl of 50 mM cellobiose in 50 mM acetate buffer pH 5.0 was serially diluted two-fold in 50 mM acetate buffer pH 5.0 in an 8-well PCR strip tubes per enzyme. To measure background glucose levels of the substrate, a strip without enzyme was prepared with each assay. The enzymes prepared as above were transferred to a pre-warmed tube strip with serial dilutions of cellobiose at once using a multi-channel pipette, and incubated for 3 minutes. The reaction was stopped by immediately placing the tube strip at 100°C for 3 minutes and cooled on ice. 10 µl of each reaction was transferred to a 96-well plate, and 190 µl of glucose assay reagent (Sigma G3293) was added along with glucose standards. The plate was incubated at room temperature for 30 min, and read at 340 nm in the plate reader.

#### c) MUG in-gel assay

One μg/well of partially purified BGLs were loaded onto wells of a 7% native polyacrylamide gel. The samples were run in 1× Tris-glycine gel running buffer at 80 kV until the loading front reached near the end of the gel. The gel was then fixed in 20 ml 25% isopropanol in 50 mM acetate buffer pH 5.0 at room temperature for 20 minutes, and incubated in 20 ml 200 µM MUG (Sigma M9766) in 50 mM acetate buffer pH 5.0 for 10 minutes with shaking at RT, then at 37°C with occasional manual shaking for 20 minutes. The gel was then visualized using trans-UV illumination in a Bio-Rad Gel Doc gel imaging system and digitally photographed.

### Endo-H treatment of BGLs

P. pastoris- produced, secreted rD2 in was readily detected in culture supernatants by BGL activity assays and Western blotting against hepta-histidine epitope in the culture supernatant of the *P. pastoris* strain, but not from a wild type control strain (Figure S1 in File S1). For native proteins, 1 µl of BGLs were treated with 0.5 µl of EndoH in 1× G buffer (enzyme and buffers, New England Biolabs P0703S) at 37°C for 3 hours.

### Protein quantification

Five μl of samples were serially diluted in duplicates along with serially diluted protein standard in triplicates, and protein concentration was measured by adding protein assay reagent (Bio-Rad 500–0006) according to the user manual.

### Protein identification of nD2 variants

Concentrated size exclusion chromatography fraction pools were run on a 7% SDS polyacrylamide gel and stained with SYPRO Ruby protein stain overnight, and photographed using Gel Doc system (Bio-Rad). Based on previous samples, gel pieces corresponding to MUG activity were excised from the gel and sent to Proteomics & Mass Spectrometry Facility at Donald Danforth Plant Science Center (St. Louis, MO) for amino acid identification using a LTQ Orbitrap Velos mass spectrometer (Thermo Scientific). Scaffold 3 proteome software (http://www.proteomesoftware.com/Proteome_software_prod_Scaffold.html) was used to annotate and view results.

### Release of *O*-linked glycans

The samples were dialyzed against Nanopure water at 4°C overnight using Tube-O-dialyzer (MW cut off 4000, G Biosciences) and the contents in the tubes were dried in a vacuum concentrator. The *O*-linked carbohydrate fractions were cleaved from the sample by β-elimination procedures [33]. Briefly, 1 M sodium borohydride in 50 mM sodium hydroxide (NaOH) was added to the samples and incubated overnight at 45°C. The incubated samples were neutralized with 10% acetic acid and desalted by passing through a packed column of Dowex™ resins (50 W × 8–100, H^+^ form, Sigma Aldrich, St. Louis,MO) and lyophilized. The borate was removed as methyl borate. The *O*-glycans were separated from residual material by passage through a C18 reversed phase cartridge. The carbohydrate fractions (*O*-linked glycans) were eluted with 5% acetic acid. The 5% acetic acid fractions were further washed with ethyl acetate and dried by lyophilization. The *O*-glycan fraction thus obtained were permethylated based on the method of Anumula and Taylor [34] and profiled by mass spectrometry. *O*-glycan analyses were performed by the Complex Carbohydrate Research Center at University of Georgia, Athens, GA.

### Mass spectrometry

MALDI/TOF-MS was performed in the reflector positive ion mode using α-dihyroxybenzoic acid (DHBA, 20 mg/mL solution in 50% methanol: water) as a matrix. The spectrum was obtained by using a Microflex LRF (Bruker).

NSI-MSn analysis was accomplished by using a LTQ Orbitrap XL mass spectrometer (Thermo Scienfitic) equipped with a nanospray ion source. Permethylated glycans from each sample were dissolved in 1 mM NaOH in 50% methanol and infused directly into the instrument at a constant flow rate of 0.5 µl/min. A full FTMS spectrum was collected at 30,000 resolution with 3 microscans. The peak intensities of each O-glycan component were obtained by averaging the 30 full FTMS scans for each sample. The capillary temperature was set at 210°C and MS analysis was performed in the positive ion mode. For total ion mapping (automated MS/MS analysis), m/z range, 300 to 2000 was scanned with ITMS mode in successive 2.8 mass unit windows that overlapped the preceding window by 2 mass units.

## Results

### An indigenous Taiwanese fungus *C. raphigera* strain *D2* expresses a strongly active β-glucosidase, D2, and possesses a single gene encoding the BGL

**Figure 1:**
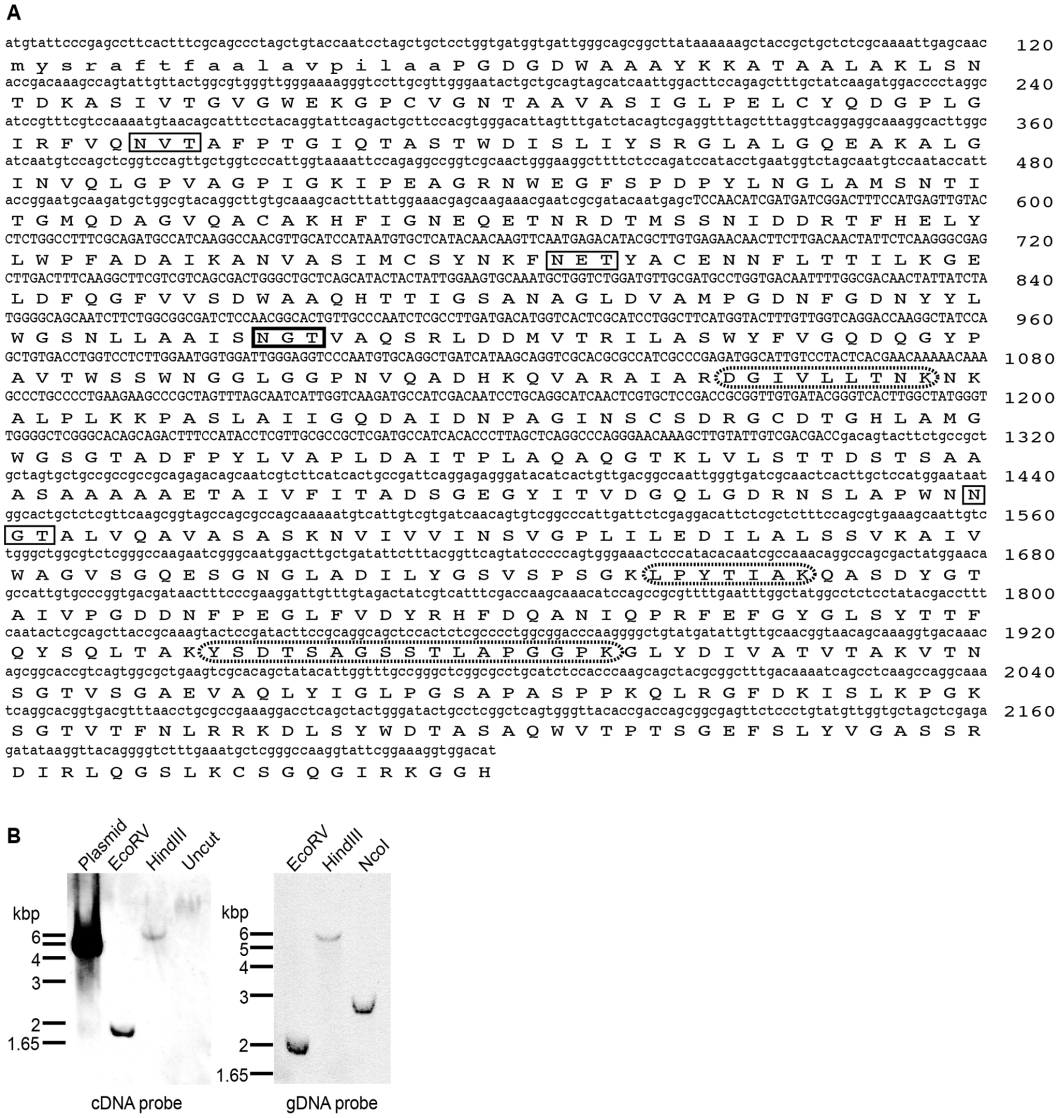
*C. raphigera* β-glucosidase, D2, is encoded by a single gene. (A) cDNA and amino acid sequences of *C. raphigera* D2. Upper case DNA sequence indicates the region used as a Southern blotting probe (738–1025bp). Amino acid sequence shown in lower case letters indicates putative signal peptide. Solid box, consensus amino acids for a predicted *N*-glycosylation site (box with thicker lines indicates the most probable *N*-glycosylation site). Dotted ovals, common peptides identified by MS in both large and small native D2. (B) Southern blotting of *C. raphigera* genomic DNA. Left, *C. raphigera* genomic DNA was cut with indicated restriction enzymes and probed with a PCR-amplified region using genomic DNA as a template. Right, *C. raphigera* genomic DNA was cut with indicated restriction enzymes and probed with a PCR-amplified region using cDNA as a template.

In previous work, a highly active BGL, D2 was identified from a native Taiwanese fungus *C. raphigera* strain *D2* for which cDNA sequence was not available. The N-terminal amino acid sequence of this protein was subsequently determined. The amino acid sequence was used to design PCR primers for the cloning of D2 cDNA (Figure 1; GenBank accession number KJ939445). We performed Southern blotting of genomic DNA isolated from *C. raphigera* strain *D2* and showed that there is only a single gene encoding D2-BGL within *C. raphigera* genome (Figure 1C). We then proceeded to characterize biochemical properties of D2.

### *C. raphigera* strain *D2* produces β-glucosidase activities with different molecular sizes, which were identified as a single protein

**Figure 2:**
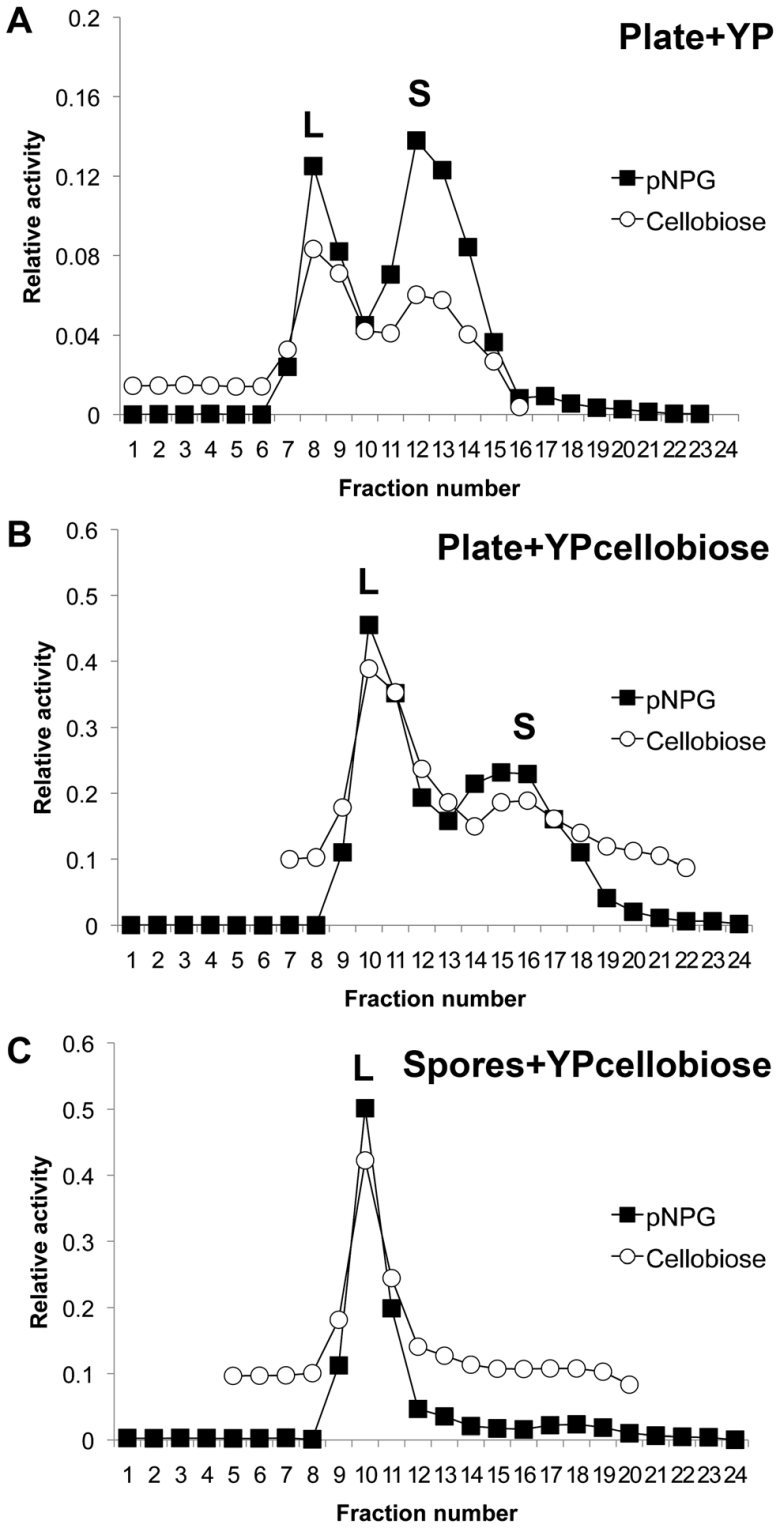
*C. raphigera* produces two variants of β—glucosidase that can be separated by size exclusion chromatography. The peak of activity recovered from anion exchange chromatography was concentrated and resolved by size exclusion chromatography. BGL activity of the fractions was monitored using pNPG (closed squares) and cellobiose assays (open circles). Two peaks, large (**L**) and small (**S**) were identified in varying amounts depending on fungal growth conditions. (A) Sephadex G200 fractions analyzed by pNPG and cellobiose assays from 3-day old YP liquid culture with 14-day old *C. raphigera* on potato dextrose agar with agar also transferred into the liquid medium. (B) From 3-day old YP liquid medium with 10 mM cellobiose with 14-day old *C. raphigera* on potato dextrose agar. (C) From YP liquid medium with 10 mM cellobiose inoculated with spores scraped from a 14-day old *C. raphigera* plate. Results were representative of three independent studies.

Upon further characterization of the native fungus, we discovered that *C. raphigera* produced two BGL activity peaks that were separable by size-exclusion chromatography but not by anion exchange chromatography; we interpreted this as proteins sharing the same isoelectric point, but with different molecular weights (Figure 2). Interestingly, depending on the growth condition of the fungus, the ratio of the peaks changed. When the *C. raphigera* was grown on potato dextrose agar (PDA) plates for 14 days and then transferred (agar blocks containing hyphae and spores in toto) to a liquid medium (YP, 1% yeast extract and 2% peptone) for three days, both peaks were present (Figure 2A). However, when the plate was transferred to YP + 10 mM cellobiose or spores scraped from 14-day old PDA plates were used to inoculate YP + glucose, the larger molecular weight peak became more prominent (Figures 2B and Figure S2 in File S1). Furthermore, when YP + 10 mM cellobiose was inoculated with spores (without hyphae and agar), the lower molecular weight peak was no longer observed (Figure 2C).

**Figure 3:**
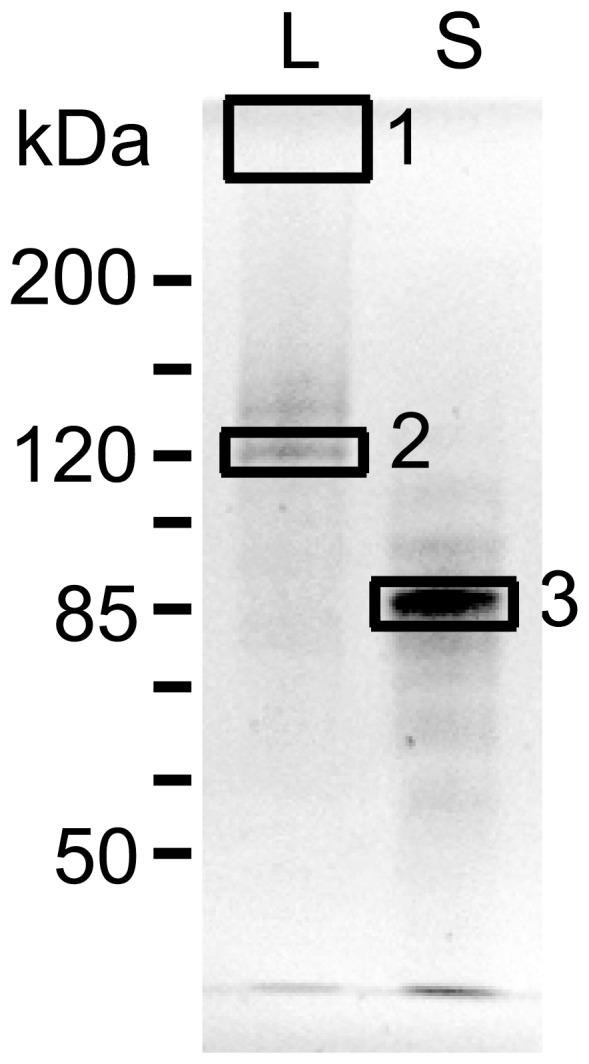
Protein bands corresponding to BGL activities identified as D2 by MS. Concentrated size exclusion chromatography fraction pools were run on a 7% SDS polyacrylamide gel and stained with SYPRO Ruby protein stain overnight, and photographed using Gel Doc system (Bio-Rad). **L**: higher molecular weight peak, **S**: lower molecular weight peak. For the actual sample submission, L and S were loaded 2 lanes apart from each other to avoid cross-contamination before cutting out the bands. None of the bands was identified with >95% probability as a known protein in NCBI database using Mascot sequence query at the time of analysis (December 2010). All 3 bands were identified as the D2 protein with 100% probability.

**Figure 4:**
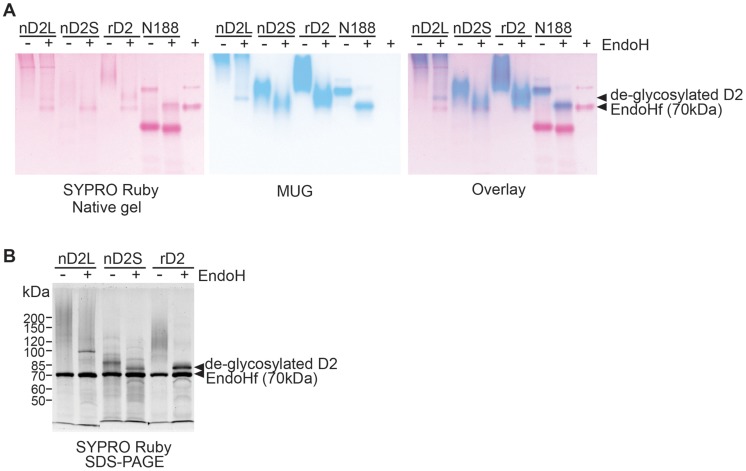
rD2 and nD2S are N-glycosylated, but nD2L has more extensive modification besides N-glycans. (A) D2 variants on a 7% native polyacrylamide gel. Left: SYPRO Ruby protein staining, middle: MUG BGL activity staining, right: merged image of MUG and SYPRO Ruby stainings. Protein ladder did not clearly separate according to a correct size pattern on a native gel and thus omitted. (B) SYPRO Ruby-staining of D2 variants, boiled and run on a 7% SDS gel, with or without EndoH treatment. PageRuler Unstained (Fermentas) was used as a size marker.

We next designed studies to establish whether both “Large” and “Small” activities originated from the same protein. To follow BGL activities on protein gels, we used 4-methylumbelliferyl β-D-glucopyranoside (MUG) staining. When protein gels are incubated with this compound, BGL activities within the gel release the fluorescent methylumbelliferone moiety of MUG, enabling detection by ultraviolet illumination [35]. To investigate whether these two activity peaks represent D2 or two distinct proteins, we excised protein bands from a SYPRO Ruby-stained SDS gel for protein identification by mass spectrometry (MS; Figure 3). Band #1 was barely visible by SYPRO Ruby staining, but the location concurred with a strong BGL activity by MUG staining of the gel (Figure 4). Interestingly, only L remained active toward MUG on SDS gels when samples were not boiled before loading, while the band in S did not. Samples were digested with trypsin, resulting peptides were separated by liquid chromatography, and analyzed by MS. 13 unique peptides from band #3 matched the amino acid sequence of native D2, and three peptides from band #2 (the matched sequences shown in Figure 1, boxed with dotted line) and two from band #1 (Table S1 in File S1). One of the peptides (DGIVLLTNK) coincided within the genomic DNA region confirmed by Southern blotting (Figure 1A).

To investigate potential post-translational modifications of native D2 proteins that would explain the observed size differences, we treated the partially purified proteins with endoglycosidase H (EndoH) to remove *N*-glycans. As references, recombinant D2 expressed in *Pichia pastoris* (rD2) and commercially available BGL, Novozyme 188 (N188, Novozymes) were also analyzed. rD2 ran as a long smear on native and SDS gels that focused to a tight band around 75.5 kDa upon EndoH treatment, suggesting that D2 expressed in *P. pastoris* is heterogeneously *N*-glycosylated. As proteins may not migrate according to their sizes on native gels, MUG- and SYPRO Ruby- stained native gel images were overlaid to indicate the location of BGL activities relative to protein bands (Figure 4A). The comparative staining of the gel images showed that most active part of nD2L was located at the top of the gel, barely entering the separating gel. After an EndoH treatment, a faster-moving, faint but focused band appeared, although most of the activity remained at the top of the gel (Figure 4A). nD2S was faster-moving than nD2L and rD2, but its electromobility increased after an EndoH treatment. rD2 had lower electromobility compared to nD2S, but it increased after an EndoH treatment (Figure 4A). This experiment also showed that N188 contained a major low molecular weight protein band by SYPRO Ruby staining that lacks BGL activity, and its BGL activity band clearly shifted down after an EndoH treatment. There was no extensive smearing of N188, indicating that *N*-glycosylation on this protein is relatively homogeneous. The SDS-PAGE followed by SYPRO Ruby staining showed that Endo-H treated nD2S and rD2 focused to the predicted size of D2 (75.5 kDa), indicating that nD2S and rD2 are *N*-glycosylated, but the majority of nD2L had modifications that alter its molecular weight in addition to *N*-glycosylation (Figure 4B).

### Structural characterization of glycan modifications of active nD2 variants

#### *O*-linked oligosaccharide analysis on the active nD2 variants by mass spectrometry

**Figure 5:**
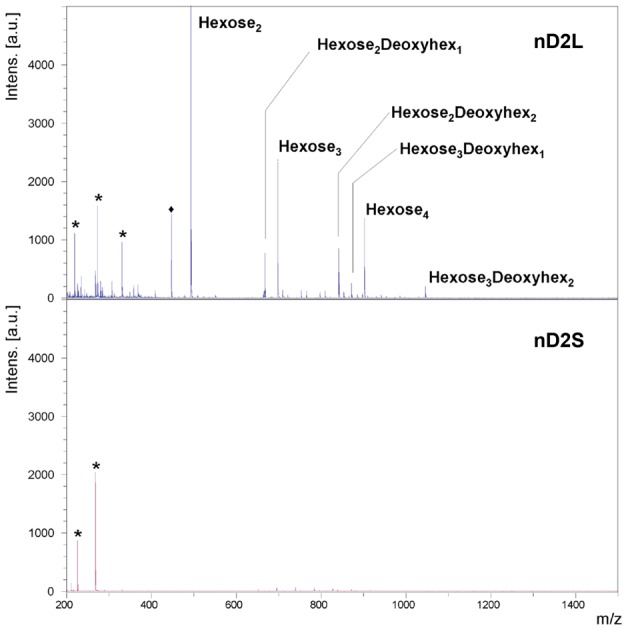
MALDI/TOF-MS spectra shows *O*-glycosylation of nD2L. *O*-linked profiling of equal amounts of nD2L and nD2s protein harvested indicated that nD2L is heavily *O*-glycosylated (A), while no *O*-glycosylation was detected on nD2S (B). Signals shown with asterisks (*) are background signals from the matrix. A signal shown with a diamond (⧫) denotes the presence of a small glycan fragment (correspond to Hexose_1_Deoxyhexose_1_, non-reduced form).

**Figure 6:**
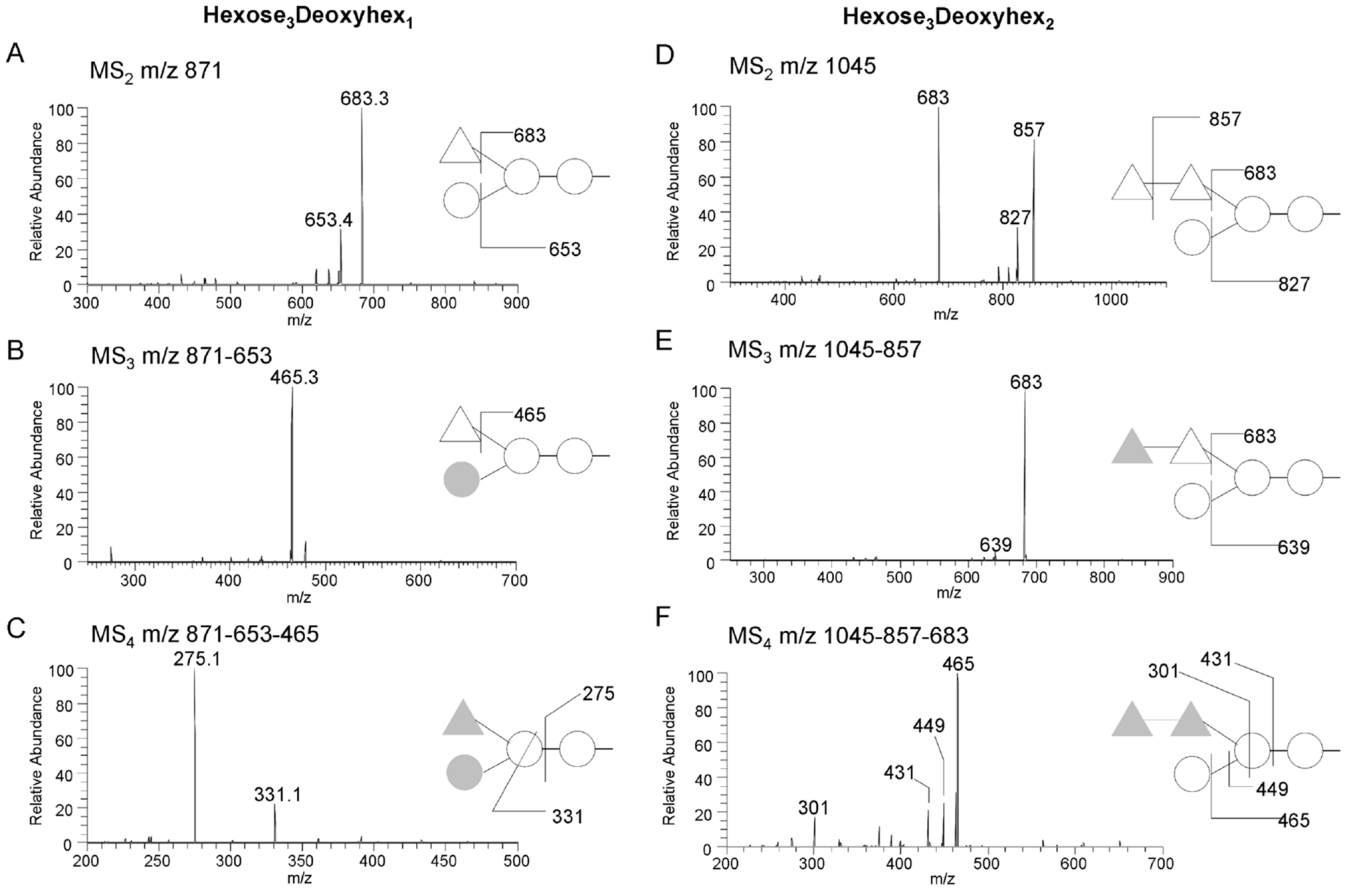
Fragmentation of deoxyhexose-containing *O*-glycans in nD2L. The main neutral loss observed in MS/MS from deoxyhexose-containing O-glycans was loss of m/z 189, indicating the deoxyhexose located at non-reducing end (A and D). The neutral loss of m/z 189 in MS2, followed by the loss of m/z 174 in MS3 in E, shows the presence of internal deoxyhexyose (m/z 174), indicating sequential terminal deoxyhexose residues. A fragment ion m/z 275 (C) and the neutral loss of m/z 252 (F) demonstrate that there is no branching at the reducing-end hexose. Triangle, deoxyhexose; circle, hexose; gray shapes indicate lost residues.

Based on our Endo-H results, we hypothesized that nD2L bore additional modifications beyond *N*-glycans. To confirm the presence of *O*-glycosylation, we subjected nD2L and nD2S to β-elimination, permethylated the released *O*-glycans from each sample, and analyzed them by Matrix-assisted laser desorption/ionization time-of-flight mass spectrometry (MALDI/TOF-MS) and by nanospray sequential mass spectrometry (NSI-MSn) (see Material and Methods). We detected strong *O*-glycan signals from nD2L, but none from nD2S (Figure 5). The *O*-glycan components observed were oligosaccharides containing two or four hexoses and zero to two deoxyhexoses. To analyze the glycan sequence in detail, we carried out MSn analysis (Figures 6 and Figure S3 in File S1), which indicated a trihexosyl chain wherein the central hexose carries a side chain of one of two deoxyhexose residues (Figure 6).

#### rD2 and nD2S have similar catalytic and biochemical properties, whereas nD2L has distinct properties

**Figure 7:**
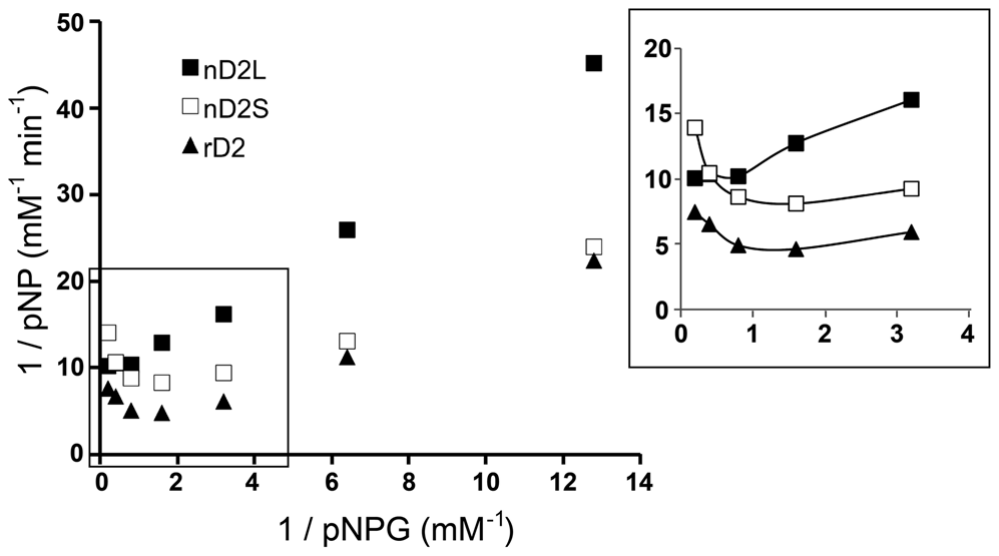
nD2L does not exhibit substrate inhibition by pNPG. pNPG assay was performed as described in Materials and Methods. The Lineweaver-Burk plot shows a substrate inhibition curve (emphasized in the inset) that indicates diminishing activity of nD2L and rD2 as substrate concentration increases.

**Figure 8:**
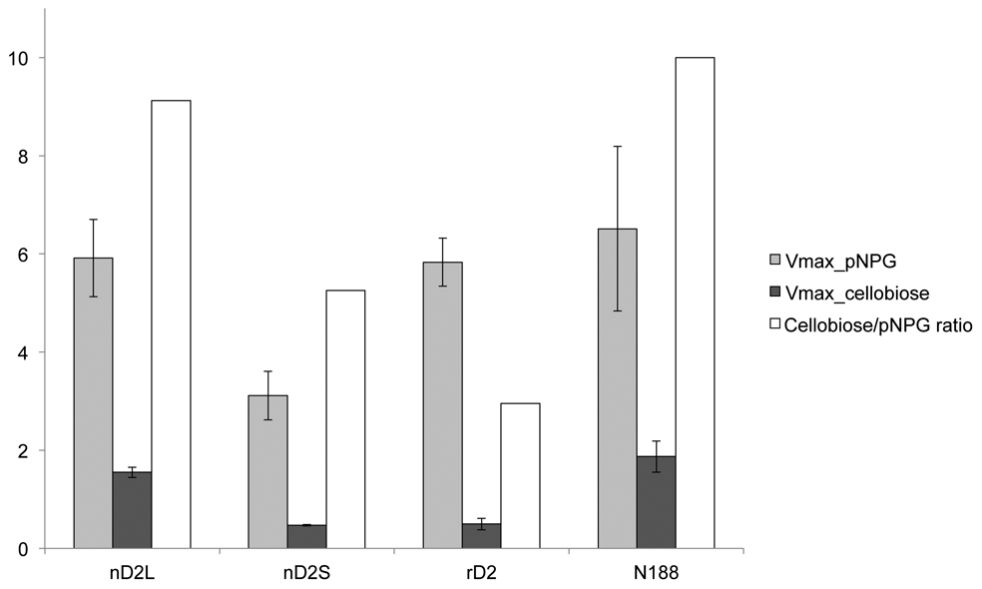
nD2L is more active toward cellobiose than small forms of D2. Maximum activity ratio of D2 variants toward cellobiose and pNPG. Activity data were complied from at least 3 independent experments. The difference between nD2L and nD2S is statistically significant (p <0.0002) by Welch's t-test. Error bars indicate standard error of the mean.

To investigate the correlation between different degrees of glycosylation and enzymatic properties, we compared catalytic properties of nD2L, nD2S and rD2. pNPG hydrolysis is a commonly used colorimetric assay for assessing BGL activities. pNPG acts as an artificial substrate, releasing *p*-nitrophenol which can be detected at the wavelength of 405 nm by spectrophotometry. First, we optimized the reaction time to one minute after monitoring pNPG activities of the BGLs at 30-second intervals at 50°C, in order to determine initial rates at various concentrations of pNPG. Both nD2S and rD2 exhibited substrate inhibition by pNPG, although nD2L did not (Figure 7). Next, we measured activities of the D2 variants on the native substrate, cellobiose, determined Km and Vmax of the BGLs for each substrate (Table 1, Figure 8). We found that the major differences among the glycovariants are the substrate inhibition by pNPG and comparative activity toward cellobiose; nD2L had a higher ratio of cellobiose to pNPG activity compared to smaller glycovariants (Figure 8, open bars).

**Table 1:** Km and Ki of partially purified BGLs.

	nD2L	nD2S	rD2	N188
pNPG Km (mM)	0.243±0.016	0.141±0.023	0.254±0.058	1.252±0.164
pNPG Vmax (μmole/sec/mg protein)	5.196±0.788	3.133±0.494	5.832±0.489	6.512±1.675
Cellobiose Km (mM)	2.314±0.185	0.783±0.122	1.186±0.208	2.527±0.506
Cellobiose Vmax (μmole/sec/mg protein)	1.550±0.105	0.470±0.014	0.495±0.119	1.871±0.318
Ki_glucose_ on pNPG	1.691±0.337	1.001±0.125	2.929±0.535	4.280 ±0.747

**Figure 9:**
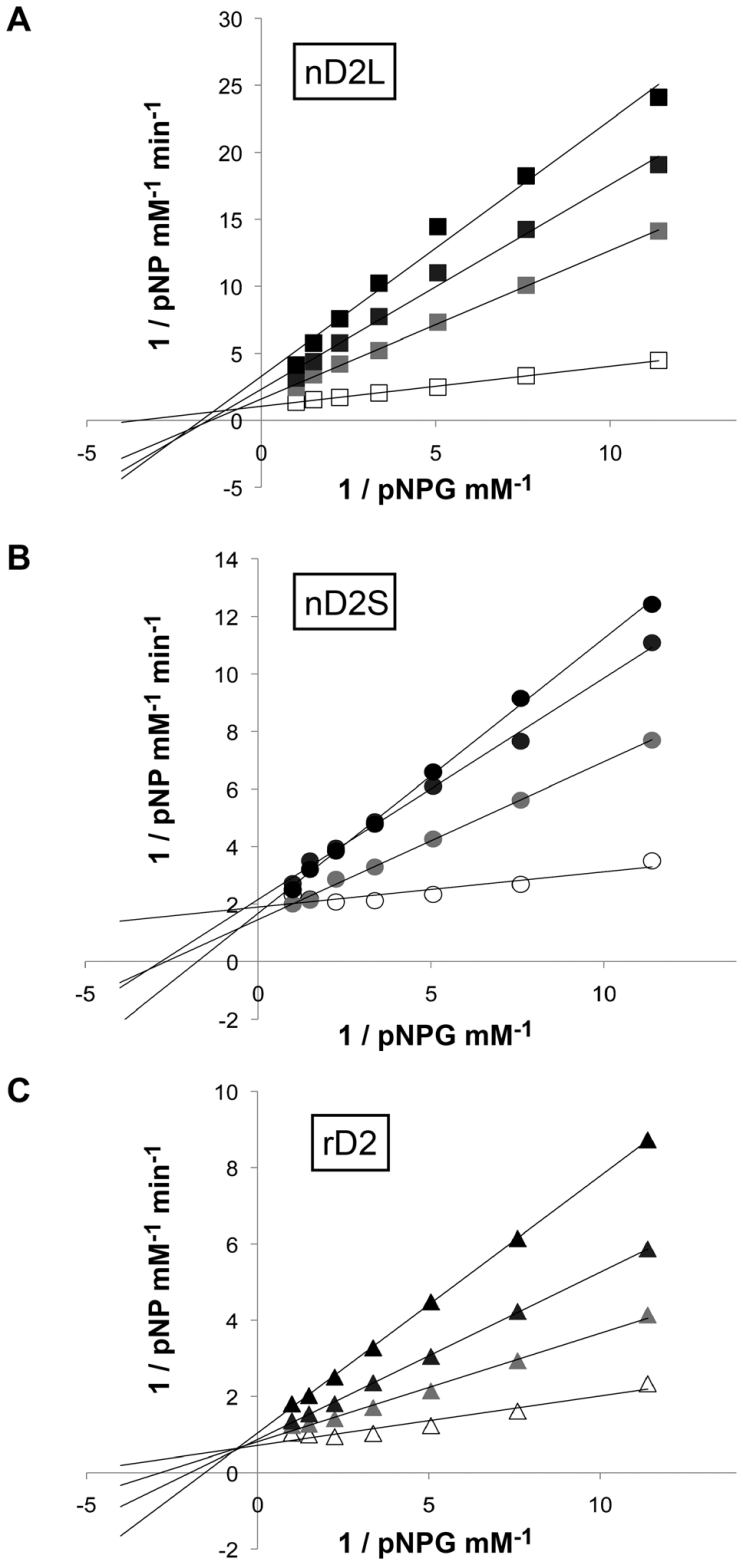
Glucose inhibition of nD2L in pNPG assay is noncompetitive. Lineweaver-Burk plots of pNPG activity of D2 variants in the presence of glucose. (A) nD2L, (B) nD2S, (C) rD2. Open symbols: 0 mM, light gray: 3 mM, dark gray: 6 mM, black: 9 mM glucose.

Glucose is a known inhibitor of BGLs, and BGLs with low glucose inhibition are desirable for commercial use in digesting cellulosic biomass [36]. Since the cellobiose assay glucose as readout, we used the pNPG assay to investigate glucose inhibition of various D2 forms. Plotting data as Lineweaver-Burk plots, it was apparent that glucose inhibition of nD2L was noncompetitive (same x-intercept, Figure 9A), whereas inhibition of nD2S and rD2 was competitive or mixed (same y-intercept or no shared intercept at either axis, Figure 9B and C). When a fixed concentration of pNPG was used as a substrate with increasing concentration of glucose, nD2S and rD2 seemed to be more tolerant of glucose (Figure S4 in File S1).

**Figure 10:**
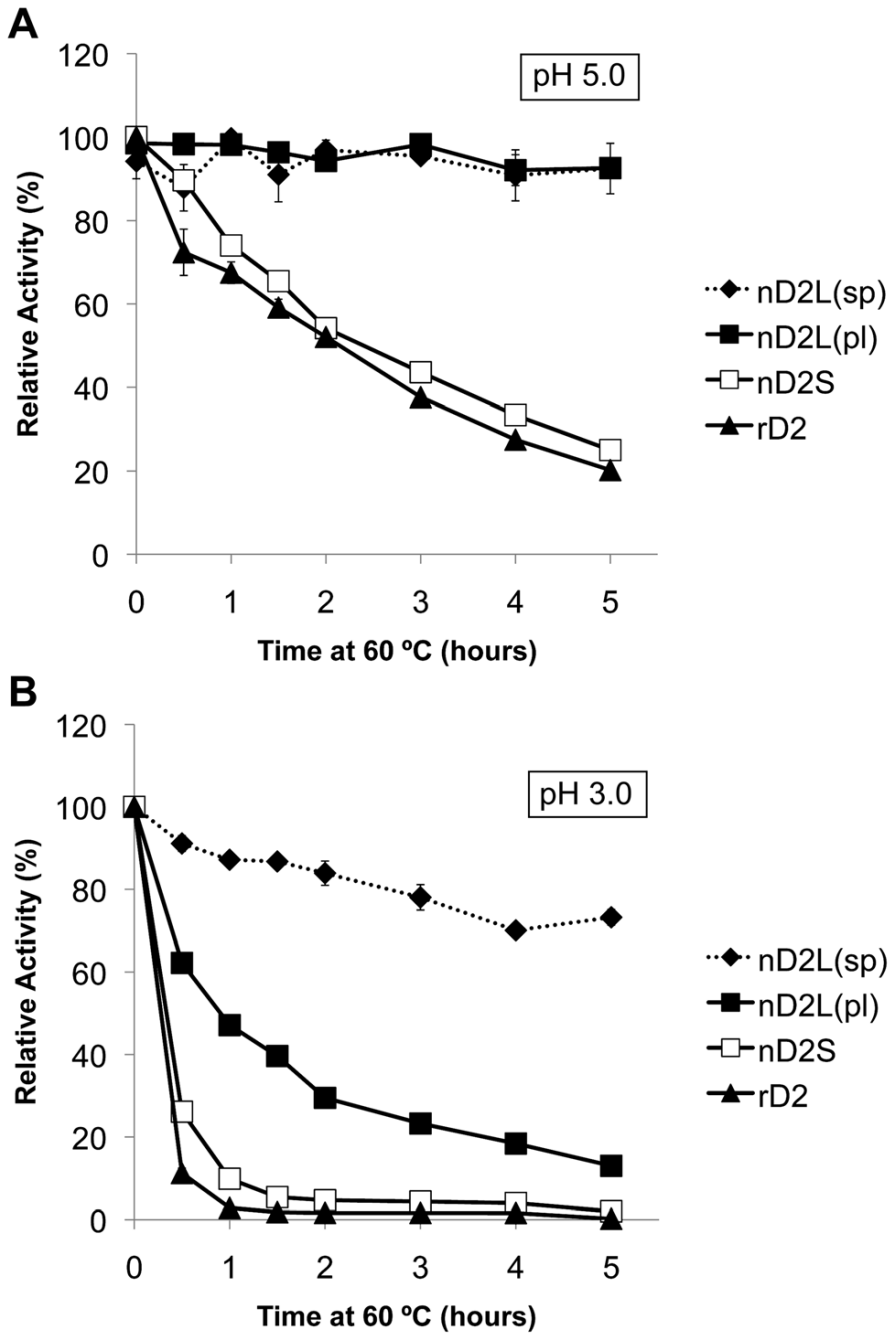
nD2L is more thermostable than smaller forms at pH 5.0, and nD2L purified from spore-inoculated culture (even more extensively glycosylated) is thermostable at pH 3.0. pNPG activity of D2 variants that were incubated at 60°C at pH 5.0 or pH 3.0 for indicated length of time. pl designates samples purified from plate-inoculated medium; sp designates samples purified from spore-inoculated medium.

After determining that catalytic properties of nD2S and rD2 differed from those of nD2L, we next compared biochemical properties of these enzymes using the pNPG assay. We found that the small forms had an optimal pH of 5.0, while nD2L was more active toward lower pH and was more sensitive to elevated pH (Figure S5 in File S1). Because it has been reported that *N*-glycosylation renders proteins more thermostable [8], [37], we investigated the thermostability of these enzymes with extensive *O*-glycosylation. In this analysis we included nD2L from spore-inoculated liquid culture (Figure 2C) and plate-inoculated liquid culture (Figure 2B), because the former exhibited even higher degrees of glycosylation (there was no marked difference in kinetic properties between spore-inoculated, hyperglycosylated nD2L (labeled as nD2(sp)) and plate-inoculated nD2L (nD2L(pl)); Figure S6 in File S1). All enzymes were incubated at 60°C at pH 5.0 (Figure 10A) or pH 3.0 (Fig. 10B) for indicated length of time before pNPG activity was measured at pH 5.0. nD2L was significantly more thermostable than the small forms at pH 5.0 (Figure 10A). nD2L(sp) and nD2L(pl) were equally stable at this pH. However, there was a drastic difference in thermostability at pH 3.0 between nD2L(sp) and nD2L(pl), indicating that extremely high degree of glycosylation protects proteins from high temperatures in acidic conditions (Figure 10B).

**Figure 11:**
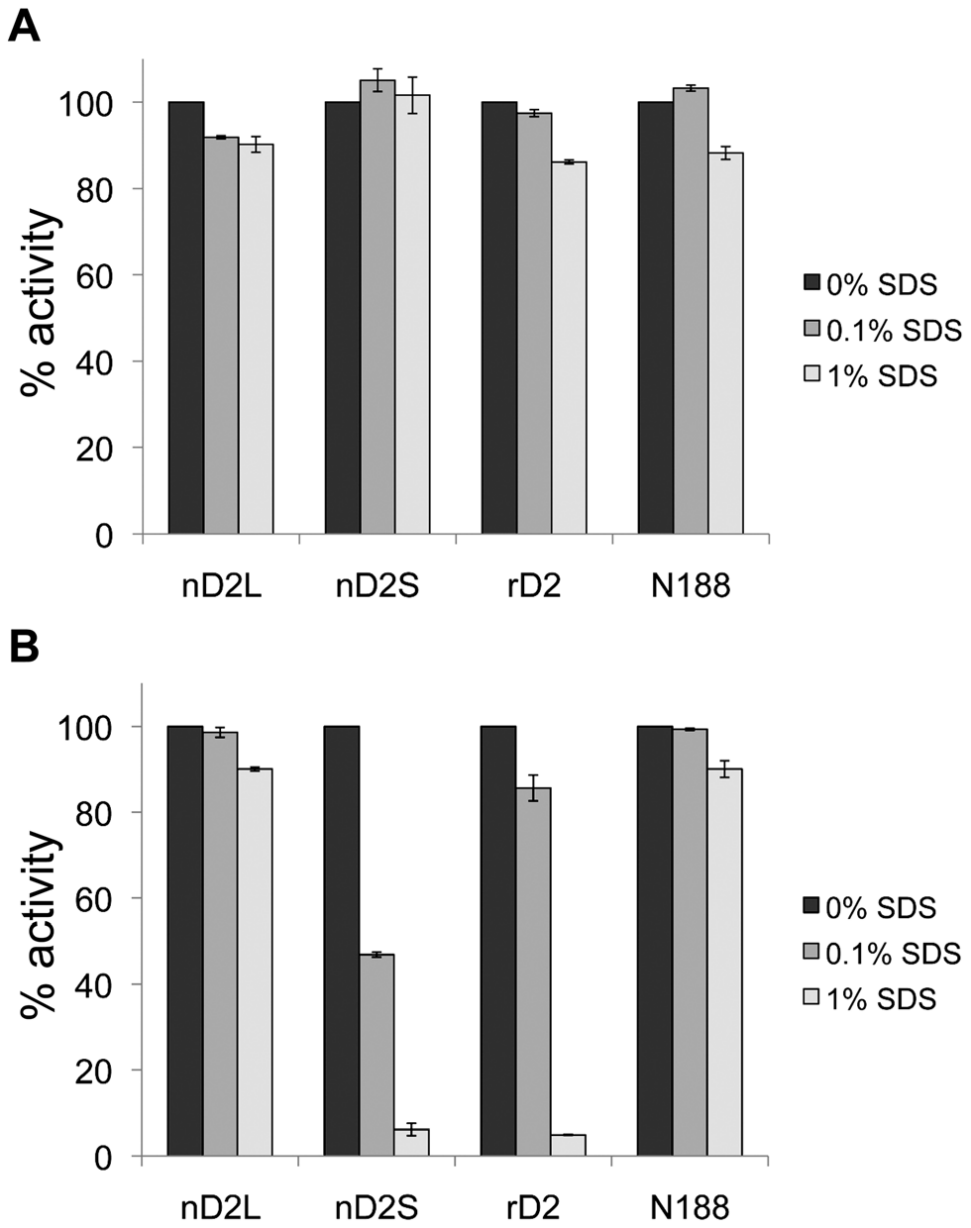
nD2L is resistant to deactivation by SDS at room temperature. Relative pNPG activity of D2 variants in the presence of SDS. (A) Enzymes assayed in the presence of SDS without the 1.5-h SDS pre-incubation prior to the assay. (B) Enzymes assayed after incubated in 1× Tris-glycine buffer with 0% (dark gray columns), 0.1% (gray columns) or 1% SDS (light gray columns) for 1.5 h at room temperature.

We noticed that we could not detect nD2S and rD2 activities by MUG staining after exposure to SDS, while nD2L and the control enzyme N188 were visible in these conditions. To investigate this phenomenon, we tested the SDS sensitivity of the BGLs using pNPG as a substrate (Figure 11). The presence of SDS in a short-term activity assay did not significantly affect BGL activity toward pNPG (Figure 11A). However, when the BGLs were incubated in conditions mimicking an SDS-gel running condition (Tris-glycine buffer, with 0.1% SDS for 1.5 hours at room temperature), nD2S activity decreased significantly (Figure 11B). With the same incubation in an elevated SDS concentration (1%), both nD2S and rD2 activities diminished, while nD2L and N188 remained active (Figure 11B, lightest gray columns), indicating that nD2L is resistant to denaturation by SDS.

## Discussion

In this study, we demonstrated that *C. raphigera* naturally produces multiple glycosylation variants of a highly active BGL, D2. The smaller molecular weight variant, nD2S, was similar in catalytic and biochemical properties to a recombinant D2 (rD2) produced in *P. pastoris*. When *N*-glycans were removed from nD2S and rD2 by EndoH treatment, each of them formed a distinct band around 75.5 kDa, the predicted size of D2. D2 has four potential *N*-glycosylation sites, with one being the most probable (Figure 1A). However it is likely that all the potential asparagine residues were *N*-glycosylated to produce the slowest migrating activity on the gel, as the apparent size shift of rD2 by EndoH treatment was up to 50 kDa (Figure 4). As a typical *N*-glycan of *P. pastoris* is several kDa [38], it is possible that non-canonical sites were also utilized [39]. Because rD2 was not a distinct band before *N*-glycan removal, *N*-glycosylation of D2 in *P. pastoris* seemed heterogeneous in nature (Figure 4). Regardless of the extent of *N*-glycosylation, rD2 exhibited strong substrate inhibition toward pNPG. In contrast, nD2L did not show substrate inhibition by pNPG, and had a higher cellobiose-to-pNPG activity ratio than smaller forms of D2 (Figure 8), indicating that nD2L is more suitable for digesting cellobiose than rD2 and nD2S (summarized in Table 2). The proteins were only partially purified and extensively glycosylated to a degree that interfered with protein concentration assays. Because of this problem, obtaining accurate protein concentrations for nD2L was challenging. We suspect true Vmax values of nD2L could be higher than what we report here, although it should not affect our observation of activity ratios toward different substrates.

**Table 2:** Summary of enzymatic properties of D2 variants.

	rD2	nD2S	nD2L
*Description*	Recombinant D2 expressed in *P. pastoris*	Native D2, small form	Native D2, large form
*Molecular weight*	100-120kDa	85kDa	>150kDa*
*N-glycosylation*	+	+	+
*O-glycosylation*	−**	−	+
*Cellobiase/pNPGase activity ratio*	0.30	0.53	0.91
*pNPG substrate inhibition*	+	+	−
*Glucose inhibition of pNPGase activity*	Competitive	Competitive/mixed	Noncompetitive
*Thermostability*	−	−	+
*pH optimum*	5	5	<2
*SDS resistance*	−	−	+

*>200 kDa for spore-inoculated nD2L.

**Not confirmed by MS, but not a significant contribution to size shift.

Glycosylation of proteins in general is thought to render proteins more resistant to degradation, but involvement of *O*-glycosylation for this effect has been less studied [21], [22], [40], [41]. Our data suggest that *O*-glycosylation of D2 is involved in substrate specificity, substrate inhibition, and optimal pH, implicating that they bind to a substrate in a different manner. Small forms of D2 exhibited significant substrate inhibition by pNPG, and we used a simple substrate inhibition formula to calculate the Km (see Equations in File S1). It is possible that pNPG digestion reaction by small D2 forms is more complicated, such as multiple binding pockets involving multiple dissociation constants [42], although it is beyond the scope of this study to decipher the exact nature of substrate inhibition of D2. Biochemically, *O*-glycosylation of D2 seems responsible for increased thermostability, lowered optimal pH, and increased resistance to denaturation by SDS. Our results are consistent with the increased stability observed in synthetically *O*-glycosylated modules [29].

Our oligosaccharide analysis showed nD2L is both *N*-glycosylated and extensively *O*-glycosylated. Commonly, *N*- and *O*-glycans on fungal glycoproteins are mannan-like structures [43]. Fungal *N*-glycans contain large mannan chains at non-reducing ends, and some mannoses in the mannan chains could be replaced with galactofuranoses [44]–[47]. On the other hand, fungal *O*-glycans usually consist of *O*-mannosyl oligosaccharides. In this study, our data indicated that the *O*-glycans of nD2L comprise oligosaccharides containing two to four hexoses and contains zero to two deoxyhexoses as side chain(s) (Figure 6). Although deoxyhexose is often observed in glycoproteins in vertebrates, invertebrates, and plants, deoxyhexose in fungal glycoproteins were only recently reported; *N*- and *O*-glycans containing one to six deoxyhexose residues were detected in the fruiting bodies of several mushrooms [48]. The size and glycosyl composition of the deoxyhexose-containing *O*-glycans we found in nD2L sample were quite similar to those in this report, although our MSn analysis suggests distinct glycosyl sequences of the *O*-glycans (Figure 6).

We have also observed that the growth conditions of *C. raphigera* significantly affected the ratio of nD2L to nD2S, although the causes for those changes remain to be investigated. Growth on glucose-rich agar medium resulted in nD2S-dominated D2 production. When liquid medium supplemented with 10 mM cellobiose was inoculated with *C. raphigera*, nD2L increased (Figure 2B), and when it was inoculated with spores scraped from agar plates, only nD2L was detected (Figure 2C). The spore-produced nD2L was even more highly glycosylated than nD2L isolated from plate-inoculated culture (Figure S6 in File S1), and was also more thermostable at pH 3.0 (Figure 10B). These results suggest that *C. raphigera* responds to its growth environment such as nutrient and water availability by controlling glycosylation. Further studies are required to elucidate the mechanisms of glycovariant regulation by *C. raphigera*.

It is important to be aware that when expressed in a different host strain, recombinant proteins (especially eukaryotic secreted proteins) will most likely have a different set of glycosylations, which may affect enzymatic or biochemical properties of enzymes. The unquestionable advantage of rD2 is that it can be produced in much larger quantities with less resource required, in a shorter amount of time. Future work of this BGL or other commercially applicable enzymes may require alternative hosts besides regular *P. pastoris*, such as engineered yeast or filamentous fungal strains [49], [50]. Our study presented here provides productive insights into future glycoengineering of commercial enzymes.

## Supporting Information

File S1 Contains the following files: **Figure S1**. Supernatant of P. pastoris X33 with or without pGAPZα-D2His6. **Figure S2**. Size-exclusion chromatography fraction profile of YP + 20 mM glucose inoculated with spores, monitored by pNPG (closed squares) and cellobiose (open circles) assays. **Figure S3**. NSI-FT full mass spectrum of permethylated released O-glycans from nD2L. **Figure S4**. Smaller forms of D2 are more resistant to higher glucose concentrations than nD2L in pNPG assays. **Figure S5**. nD2S and rD2 have almost identical pH optima at pH 5.0, while nD2L is most active in lower pH. **Figure S6**. A time course of plate- and spore-inoculated nD2 electromobility by in-gel MUG activity assay. **Table S1**. MS/MS identification of native D2 variants.
(DOCX)
