# Supplementary material for: Glycosylation Variants of a β-Glucosidase Secreted by a Taiwanese Fungus, Chaetomella raphigera, Exhibit Variant-Specific Catalytic and Biochemical Properties
Source: PLoS One. 2014 Sep 2;9(9):e106306. doi: 10.1371/journal.pone.0106306 (PMC4152272; doi:10.1371/journal.pone.0106306)
Supplement: File S1 — Contains the following files: Figure S1. Supernatant of P. pastoris X33 with or without pGAPZα-D2His6. Figure S2. Size-exclusion chromatography fraction profile of YP + 20 mM glucose inoculated with spores, monitored by pNPG (closed squares) and cellobiose (open circles) assays. Figure S3. NSI-FT full mass spectrum of permethylated released O-glycans from nD2L. Figure S4. Smaller forms of D2 are more resistant to higher glucose concentrations than nD2L in pNPG assays. Figure S5. nD2S and rD2 have almost identical pH optima at pH 5.0, while nD2L is most active in lower pH. Figure S6. A time course of plate- and spore-inoculated nD2 electromobility by in-gel MUG activity assay. Table S1. MS/MS identification of native D2 variants. (DOCX) [file pone.0106306.s001.docx]

Yoneda *et al*., Glycosylation variants of a β-glucosidase secreted by a Taiwanese fungus, *Chaetomella raphigera*, exhibit variant-specific catalytic and biochemical properties

Supporting information


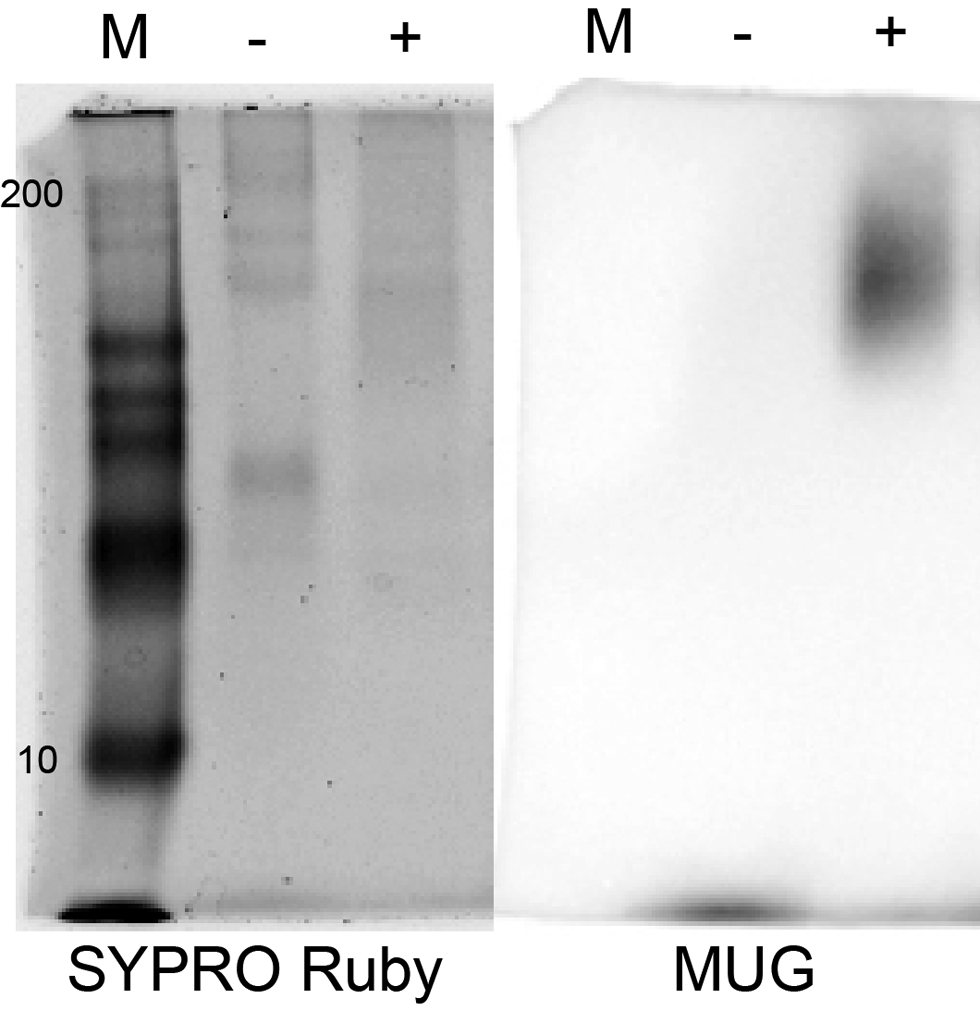


**Figure S1**. Supernatant of *P. pastoris* X33 with or without pGAPZα-D2His6.

25 µl of supernatants from P. pastoris cultures with or without heptahistidine-tagged rD2-expressing plasmid were loaded onto 7% native polyacrylamide gel, MUG activity was photographed and stained with SYPRO Ruby to visualize proteins. M, PageRuler Unstained protein marker. -, without pGAPZα-D2His6. +, with pGAPZα-D2His6.


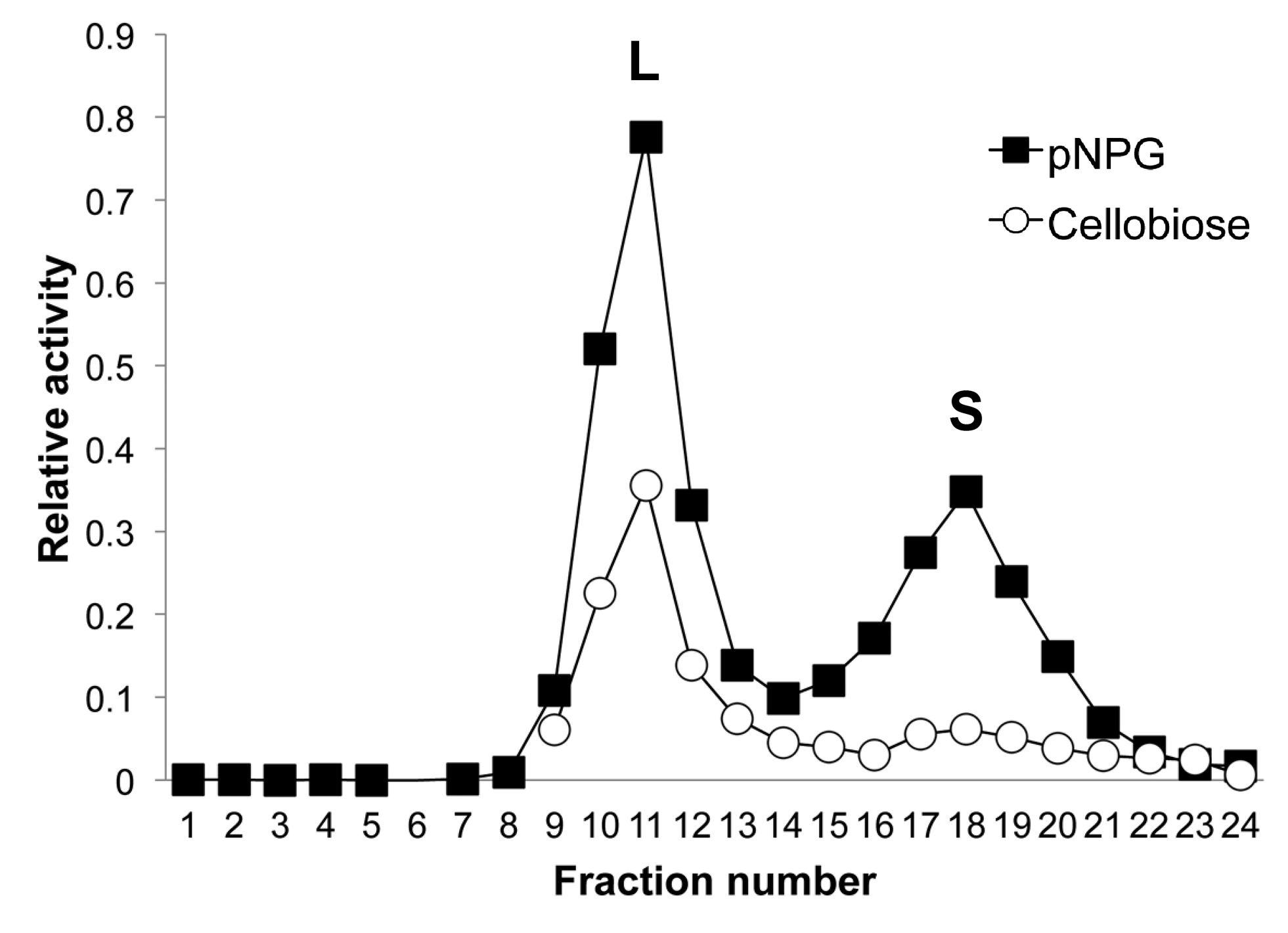


**Figure S2**. Size-exclusion chromatography fraction profile of YP + 20mM glucose inoculated with spores, monitored by pNPG (closed squares) and cellobiose (open circles) assays.


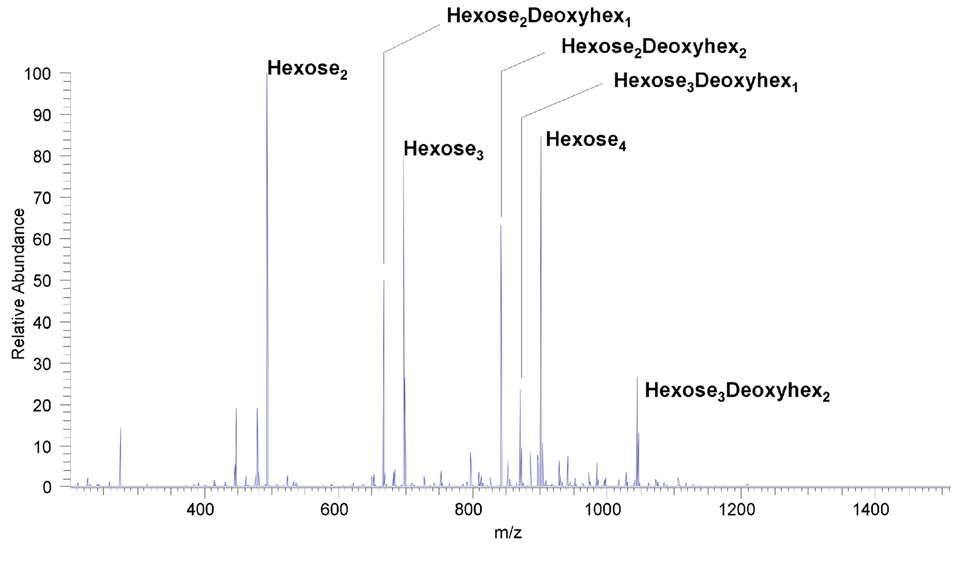


**Figure S3**. NSI-FT full mass spectrum of permethylated released O-glycans from nD2L.

Permethylated released O-glycans from nD2L were analyzed by NSI-FT-MSn. The full mass analysis was performed in 30,000 resolution and the O-glycans detected in the full mass analysis were in agreement with the MALDI/TOF-MS result shown in Figure 5.


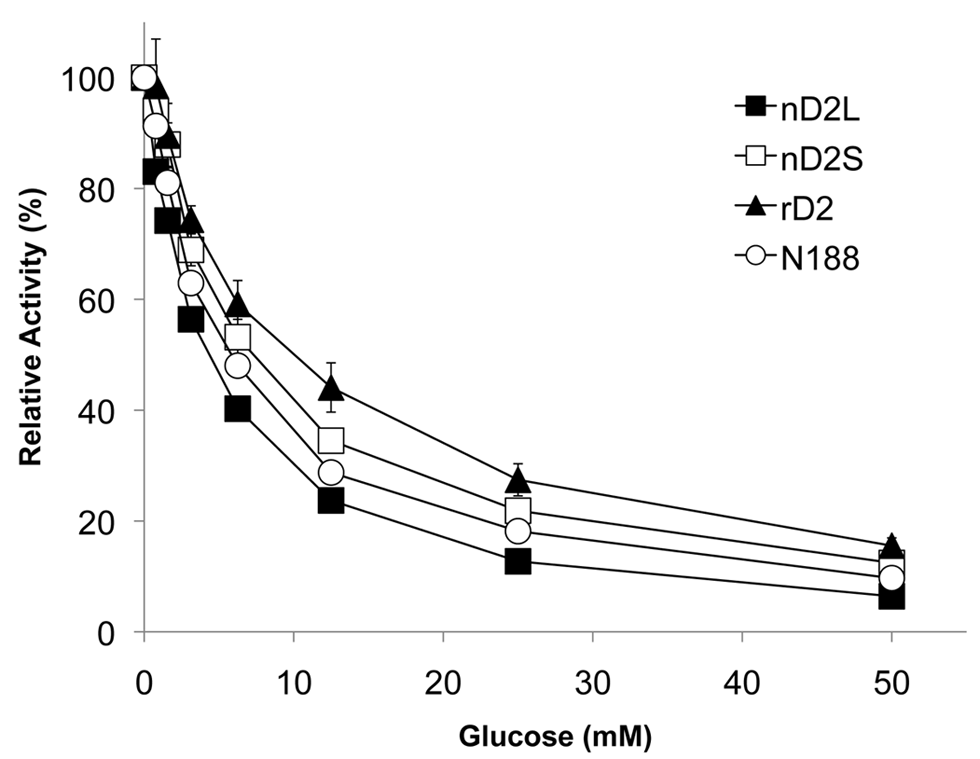


**Figure S4**. Smaller forms of D2 are more resistant to higher glucose concentrations than nD2L in pNPG assays.

Relative pNPG activity of D2 variants with 0.5 mM pNPG when 0-50 mM glucose were added.


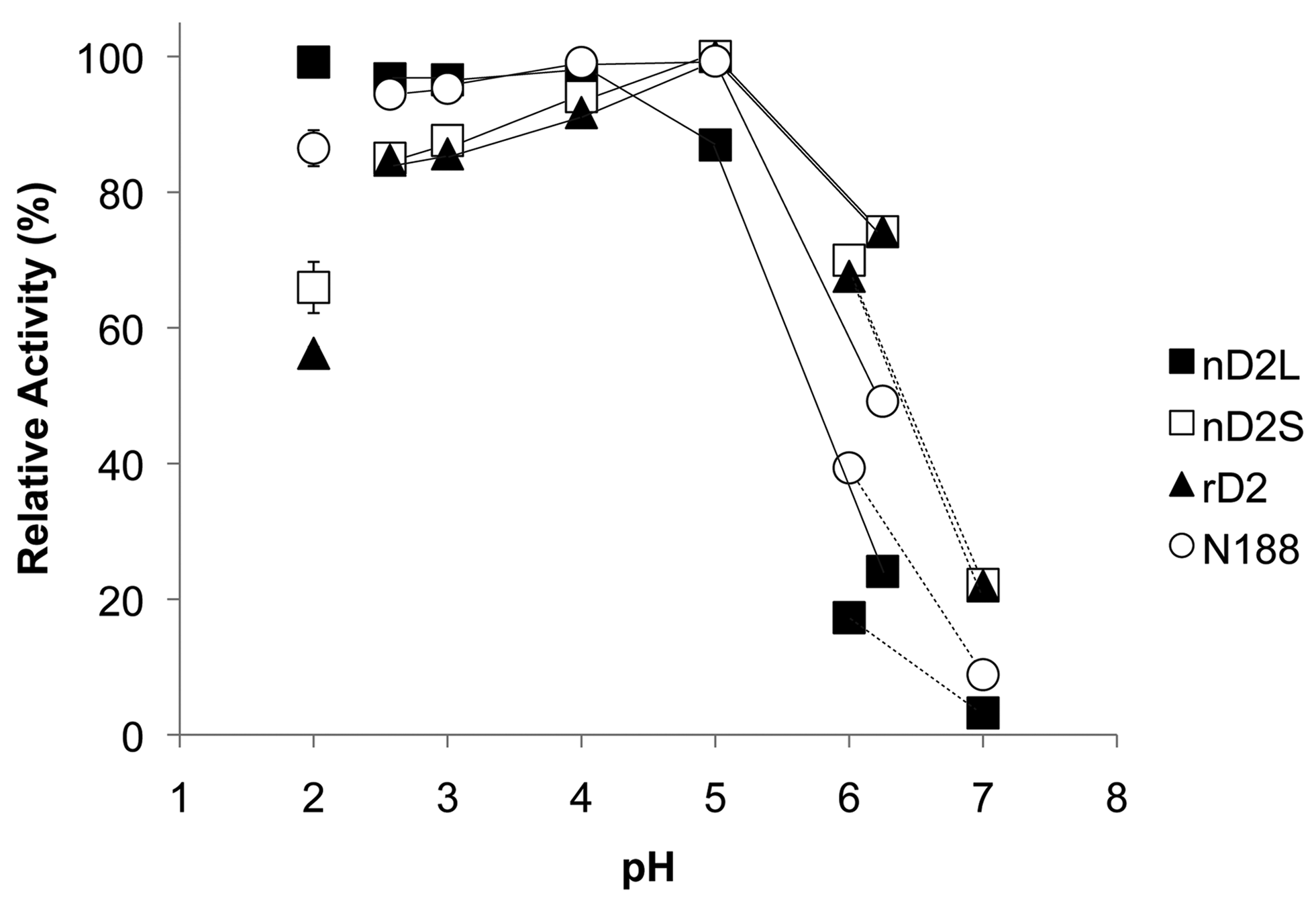


**Figure S5**. nD2S and rD2 have almost identical pH optima at pH 5.0, while nD2L is most active in lower pH.

Relative pNPG activity was plotted against pH, in 10mM HCl (pH 2.0), 50 mM acetate buffer (pH2.6-6.0, solid lines) or 50 mM phosphate buffer (pH 6.0-7.0, dotted lines).


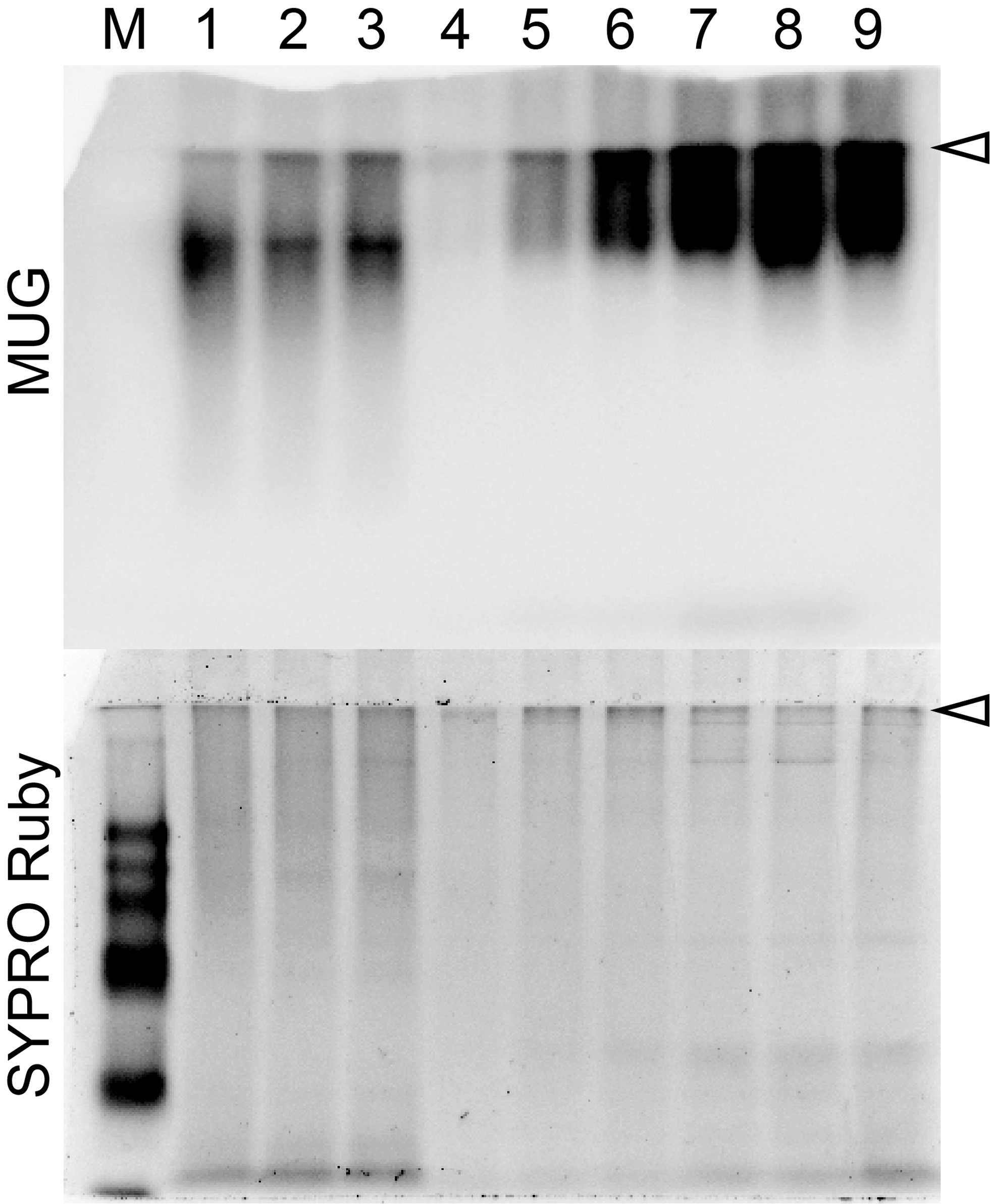


**Figure S6.** A time course of plate- and spore-inoculated nD2 electromobility by in-gel MUG activity assay. **Lane M**, Fermentas unstained ladder; **lane 1**, Plate-inoculated YP+0.2 % glucose day 2; **lane 2**, Plate-inoculated YP+0.2 % glucose day 3; **lane 3**, Plate-inoculated YP+0.2 % glucose day 4; **lane 4**, Spore-inoculated YP+0.2 % glucose day 2; **lane 5**, Spore-inoculated YP+0.2 % glucose day 3; **lane 6**, Spore-inoculated YP+0.2 % glucose day 4; **lane 7**, Spore-inoculated YP+0.2 % cellobiose day 2; **lane 8**, Spore-inoculated YP+0.2 % cellobiose day 3; **lane 9**, Spore-inoculated YP+0.2 % cellobiose day 4. Open arrowheads indicate the boarder between stacking (3.5%) and resolving (7%) native polyacrylamide gels.

| **Table S1. MS/MS identification of native D2 variants** | | | | |
| --- | --- | --- | --- | --- |
| **Sample#** | **Peptide sequence** | **ID probability** | **# of unique spectra** | **Sequence coverage** |
| 1 | DGIVLLTNK  YSDTSAGSSTLAPGGPK | 100% | 2 | 3.61% |
| 2 | DGIVLLTNK  LPYTIAK  YSDTSAGSSTLAPGGPK | 100% | 3 | 4.58% |
| 3 | ALGINVQLGPVAGPIGK | 100% | 21 | 22.2% |
|  | DGIVLLTNK |  |  |  |
|  | DIRLQGSLK |  |  |  |
|  | GFDKISLKPGK |  |  |  |
|  | GLALGQEAK |  |  |  |
|  | GLYDIVATVTAK |  |  |  |
|  | HFDQANIQPR |  |  |  |
|  | HFIGNEQETNR |  |  |  |
|  | HFIGNEQETNRDTMSSNIDDR |  |  |  |
|  | LPYTIAK |  |  |  |
|  | SGTVTFNLR |  |  |  |
|  | VTNSGTVSGAEVAQLYIGLPGSA |  |  |  |
|  | PASPPK |  |  |  |
|  | YSDTSAGSSTLAPGGPK |  |  |  |

**Equations**

Michaelis-Menten model


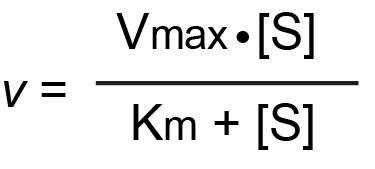


Substrate inhibition model


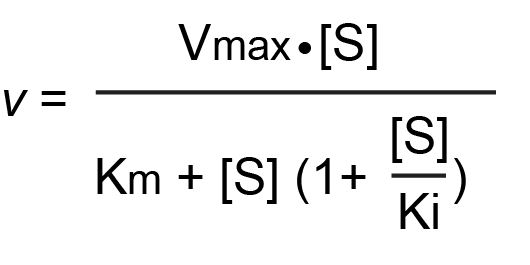


Competitive inhibition


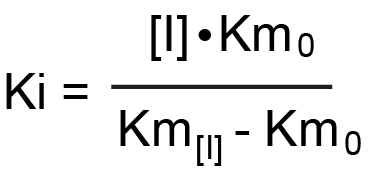


Noncompetitive inhibition


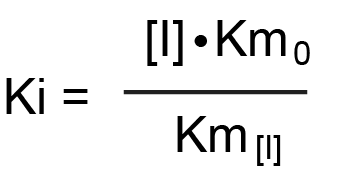


*v* = Initial velocity

[S] = Substrate concentration

Km_0_ = Original Km without inhibitor

[I] = Inhibitor concentration

Km_[I]_ = Km at inhibitor concentration [I]
